# Peer Review of “Dental Tissue Density in Healthy Children Based on Radiological Data: Retrospective Analysis”

**DOI:** 10.2196/62676

**Published:** 2024-06-20

**Authors:** Anonymous

**Keywords:** density, teeth, tooth, dental, dentist, dentists, dentistry, oral, tissue, enamel, dentin, Hounsfield, pathology, pathological, radiology, radiological, image, images, imaging, teeth density, Hounsfield unit, diagnostic imaging

*This is the peer-review report for ”Dental Tissue Density in Healthy Children Based on Radiological Data: Retrospective Analysis.”*

## Round 1 Review

### General Comments

The subject is interesting. The densities of dental hard tissues were determined by cone-beam computed tomography (CBCT), a technique that has been recently used for this purpose.

#### Minor Comments

1. The article [1] specifies the aim and is structured according to the journal’s recommendations.

2. The *Introduction* could be improved by adding some data regarding the hard structures analyzed and an appreciation of the clinical relevance of the method.

3. In the *Methods* section, the authors could compare the densities of hard tissues in the same patient by groups of teeth depending on the period of tooth bud formation.

4. The conclusions should be improved with a statement regarding the importance of the method for current practice and for its automatic use.

## Abbreviations

CBCT: cone-beam computed tomography
